# Advanced Strategies in Phage Research: Innovations, Applications, and Challenges

**DOI:** 10.3390/microorganisms13081960

**Published:** 2025-08-21

**Authors:** Pengfei Wu, Wanwu Li, Wenlu Zhang, Shasha Li, Bo Deng, Shanghui Xu, Zhongjie Li

**Affiliations:** Microbial Pathogen and Anti-Infection Research Group, School of Basic Medicine and Forensic Medicine, Henan University of Science and Technology, Luoyang 471003, China; wpf4320792@163.com (P.W.); liwanwu100@163.com (W.L.); z907733270@163.com (W.Z.); lishasha_2018@163.com (S.L.); deng_bo888@163.com (B.D.); xushanghui1208@126.com (S.X.)

**Keywords:** bacteriophages, artificial intelligence, CRISPR, phage therapy, phage-based biosensor, biocontrol

## Abstract

The escalating global threat of antimicrobial resistance (AMR) underscores the urgent need for innovative therapeutics. Bacteriophages (phages), natural bacterial predators, offer promising solutions, especially when harnessed through advances in artificial intelligence (AI). This review explores how AI-driven innovations are transforming phage biology, with an emphasis on three pivotal areas: (1) AI-enhanced structural prediction (e.g., AlphaFold); (2) deep learning functional annotation; (3) bioengineering strategies, including CRISPR-Cas. We further discuss applications extending to medical therapy, biosensing, agricultural biocontrol, and environmental remediation. Despite progress, critical challenges persist—including high false-positive rates, difficulties in modeling disordered protein regions, and biosafety concerns remain. Overcoming these requires experimental validation, robust computational frameworks, and global regulatory oversight. AI integration in phage research is accelerating the development of next-generation therapeutics to combat AMR and advance engineered living therapeutics.

## 1. Introduction

Bacteriophages (phages), the most abundant biological entities on Earth, exert profound influence on microbial ecosystems through complex interactions with bacterial hosts [1,2]. Recent progress in phage biology has been significantly driven by the ongoing antimicrobial resistance (AMR) crisis and rapid advancements in genomic sequencing technologies. Discovered in the late nineteenth century, bacteriophages remained enigmatic until next-generation sequencing (NGS) ushered in a profound expansion of our understanding of their genomic architecture and infection mechanisms [1,2,3]. A comprehensive genomic analysis involving 627 geographically diverse phages targeting a single bacterial species revealed that bacteriophages’ genetic diversity spans a continuum rather than discrete categories, and their genomes display pronounced mosaicism, affirming that phage communities possess an open gene pool perpetually enriched by foreign genes [4]. Extensive metagenomic surveys have unveiled extraordinary phage diversity, illuminating regulatory mechanisms of lytic cycles, determinants of host specificity, and lysogenic integration processes [5,6]. However, approximately 65% of phage genes defy conventional functional annotation [7], representing an immense reservoir of genomic “dark matter” with the potential to yield novel antimicrobial agents [8]. Translating these genomic insights into clinical interventions is impeded by technical challenges in precise functional prediction and by safety concerns related to horizontal gene transfer—particularly the dissemination of antibiotic-resistance determinants [9,10,11,12]. Overcoming these annotation gaps necessitates an integrated approach encompassing computational modeling, rigorous experimental validation, and robust ethical governance frameworks. However, traditional bioinformatics methods fall short in large-scale functional prediction, requiring machine learning-based innovations to overcome these limitations.

Artificial intelligence (AI) is revolutionizing the decoding of phage genomic “dark matter”. By leveraging large-scale genomic datasets, AI models now excel at host prediction, life cycle classification, and identification of antimicrobial candidates. These models not only guide the assembly of therapeutic phage cocktails but also accelerate phage characterization [13,14,15], laying the groundwork for precision phage therapeutics. Structure prediction platforms such as AlphaFold and OpenFold have resolved previously uncharacterized proteins, including endolysins and tail fibers, enabling residue-level engineering [16,17]. Such developments enable comprehensive mappings linking genomic sequences, protein structures, and bactericidal mechanisms. AI-driven molecular docking and deep learning annotation now pinpoint phage protein–host receptor interfaces and steer the design of ultra-specific lysins [18,19,20,21]. Concurrently, AI-guided CRISPR/Cas editing is crafting chimeric and synthetic phages with novel antimicrobial traits. However, rigorous experimental validation remains essential due to persistent false-positive predictions.

**Figure 1:**
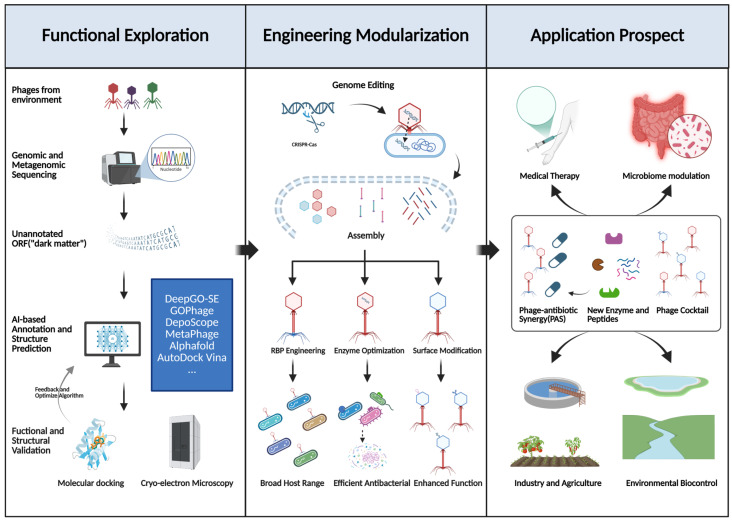
This figure illustrates the integrated research-to-application pipeline for phage engineering. It encompasses: (1) mining environmental phage resources and utilizing AI-driven advanced bioinformatic analyses combined with experimental validation to elucidate the functions of “dark matter” within phage genomes; (2) applying synthetic biology approaches, particularly CRISPR-based gene editing, for the rational design and modular engineering of phages; and (3) leveraging the engineered phages for applications aimed at combating antibiotic resistance, modulating microbiomes, as well as for industrial, agricultural, and environmental biocontrol purposes.

The convergence of computational predictions and experimental methodologies is reshaping phage engineering strategies. Cutting-edge techniques, including cryo-electron microscopy and single-molecule sequencing, provide atomic-level insights into phage biology, guiding the rational redesign of receptor-binding proteins (RBPs) and modular genome engineering [22]. Machine learning models can optimize the design of synthetic phage strains or phage consortia. This enables precise targeting of multidrug-resistant pathogens, elimination of persistent biofilms, and modulation of microbiome compositions for specific therapeutic goals [23]. Here, we first summarize advances in phage genomics and annotation technologies, explore AI-enabled progress in structural and functional prediction, analyze engineering strategies with therapeutic and other applications, and conclude with emergent challenges and future directions (Figure 1).

## 2. Advances in Phage Genomics and Bioinformatics

### 2.1. High-Throughput Sequencing and Hybrid Assembly

High-throughput sequencing and hybrid assembly have revolutionized phage genomics. Short-read Illumina platforms still offer the highest per-base accuracy and throughput rates, while long-read technologies such as PacBio HiFi and Oxford Nanopore provide reads capable of spanning long repeats, genome termini, and epigenetically modified regions that are typically inaccessible to short reads alone. Combining the two in hybrid assemblies has become standard practice, routinely producing near-complete phage genomes with >99% consensus identity [1]. Notably, this approach has enabled the recovery of jumbo phages such as *Klebsiella* phage vB_KquU_φKuK619 [24]. Building on this foundation, a hybrid, multi-polishing pipeline that combines Nanopore and Illumina reads was developed to improve assembly integrity and gene annotation accuracy [25]. Likewise, incorporating Nanopore long reads into metagenomic assemblies markedly improves viral and microbial genome recovery—a result corroborated in complex human gut viromes [26,27].

Metagenomic surveys have further expanded the known phage repertoire in understudied biomes such as marine ecosystems and the human gut [28,29,30]. Applied to Arctic metagenomes, MetaViralSPAdes uncovered three novel Asgard archaeal virus families [31], underscoring its power to resolve divergent viral clades that elude traditional assembly strategies, particularly in extreme or ancient biomes. Recent studies refined this approach by coupling MetaViralSPAdes with viralComplete, a post-assembly module that enriches viral genome quality within metagenomic datasets [32,33]. This workflow reliably identifies uncultivated viral genomes even in low-biomass or highly diverse samples, enabling broader ecological and functional insights.

Complementing DNA-based approaches, direct RNA sequencing offers a single-molecule view of the transcriptome, capturing dynamic base modifications (e.g., m^6^A) during the lytic cycle and unveiling regulatory layers invisible to DNA-centric methods [34,35,36,37]. Collectively, these innovations deepen our insights into phage diversity, regulation, and evolution across both conventional and extreme microbial habitats.

### 2.2. Integrated Annotation Pipelines

Precise gene prediction and functional annotation in phage genomes increasingly hinge on hybrid workflows that fuse classical homology-based methods—such as BLAST 2.17.0 and HMMER 3.4—with state-of-the-art machine learning and deep learning frameworks. These integrative strategies adeptly address the intricacies of phage genomic architecture, particularly the detection of short, overlapping, or non-canonical open reading frames (ORFs). For example, PHANOTATE enhances small-ORF discovery by modelling phage-specific codon usage patterns, whereas DeepPhage employs convolutional neural networks to discriminate lytic from lysogenic modules within metagenomic contigs [13,38].

Recent innovations extend this paradigm. PhageScanner [39] delivers a modular, reconfigurable machine learning pipeline for annotating phage and plasmid genomes, coupling PHANOTATE-driven ORF prediction with strand-specific, frame-aware analyses via an interactive visual interface. Similarly, VirNucPro [40] pioneers viral gene annotation by integrating six-frame translation with large language models, achieving remarkable accuracy in identifying short viral sequences (300–500 bp). By combining nucleotide and amino acid information, VirNucPro significantly surpasses conventional tools such as DeepVirFinder [41] and GCNFrame [42], particularly when applied to fragmented or low-abundance datasets.

Concurrently, VirClust [43] provides a scalable, protein cluster-based framework for viral taxonomy that aligns with ICTV guidelines, promoting consistency across comparative virome studies. Benchmarks on curated phage datasets reveal that these integrated pipelines can reduce false-negative rates by up to 25% relative to homology-only approaches while keeping false-positive levels within budgets for experimental validation. Consequently, such multilayered annotation systems have become indispensable for deciphering complex viral signals in large-scale metagenomic datasets.

### 2.3. Biological Revelations Through New Approaches

Recent methodological breakthroughs have already translated into substantive biological discoveries. Long-read assemblies combined with modification calling have revealed that phage Φ29 decorates its genome with 5-carboxylcytosine (5caC) to evade host restriction systems, inspiring the search for analogous anti-restriction strategies in other phages [44]. Comparative genomics, reinforced by AI-driven predictors, has identified capsule-specific depolymerases in *Acinetobacter* phages such as PMK34 that efficiently dismantle exopolysaccharides, rendering pathogens serum-sensitive and providing novel tools to combat catheter-associated infections [45,46]. Likewise, genome-resolved analyses of *Proteus* phages RP6 and RP7 have uncovered phage-encoded enzymes with potent antibiofilm activity [47].

Transcriptomic profiling combined with refined ORF calling pipelines has shown that the temperate *Escherichia coli* phage Φ24B expresses small regulatory RNAs (sRNAs) that bind host *recA* mRNA, dampening the SOS response and enhancing phage replication under antibiotic pressure [48]. This suggests that the interplay in phage–host regulation of gene expression may be remarkably prevalent. Another study in *Yersinia ruckeri* has revealed that the RNA chaperone *Hfq* and its associated sRNAs also play critical roles in biofilm modulation and immune evasion [49]. On the synthetic biology front, rational engineering of *Dhillonvirus* genomes guided by high-confidence annotations has enabled the deletion of modification enzymes such as nmad5 or insertion of anti-Tmn proteins, allowing engineered particles to bypass Tmn-based host immunity and restore therapeutic efficacy [50].

Taken together, the convergence of high-accuracy hybrid assemblies, context-aware gene calling pipelines, and progressively richer reference databases has transformed phage genomes from fragmented contigs into nearly complete, taxonomically resolved blueprints. However, these blueprints still read like shadow maps; more than two-thirds of their predicted ORFs remain functionally orphaned, and sequence homology alone is insufficient to illuminate their roles in infection biology [51]. Decoding this genomic “dark matter” will depend on AI capable of converting sequences into reliable predictions of structures, binding networks, and molecular activity.

## 3. AI-Driven Structural and Functional Annotation

The latest wave of AI architectures—ranging from diffusion-style structure predictors to graph-based phage–host matchers—has transformed phage genomes from fragmented contigs into experimentally testable blueprints. Here, we survey AI tools across three thematic layers (Table 1), (i) structure prediction, (ii) interface modelling and functional inference, and (iii) multi-omics integration, evaluating their strengths, limitations, and outstanding gaps.

### 3.1. Structure Prediction

The ability to predict protein structures from sequences has dramatically improved with the advent of deep learning tools such as AlphaFold, which now achieves near-experimental accuracy—even for orphan phage proteins lacking detectable homologs. These advances are closing longstanding gaps in structural annotation and enabling the rational engineering of phage components for therapeutic and diagnostic applications [16,17,52].

For instance, AlphaFold modeling of phage tail fibers revealed conserved β-helix domains responsible for host receptor specificity, allowing researchers to retarget phage host ranges through rational design [53,54]. Likewise, high-confidence models of *Streptococcus* phage PlyC endolysin enabled residue-level optimization, increasing lytic activity against methicillin-resistant *S. aureus* by 3.2-fold through substitutions in the substrate binding cleft [55,56]. Recent work has also demonstrated the power of AlphaFold in modeling structurally complex or modular proteins. Studies of *Acinetobacter* phage tail fibers have shown that AlphaFold can accurately predict trimeric conformations and domain arrangements, which has proven crucial for understanding host recognition and developing phage-based antibacterials [57,58]. Complementing AlphaFold predictions with molecular dynamics (MD) simulations, such as Gaussian accelerated MD (GaMD), refines dynamic properties of flexible loops and binding regions, particularly for RBPs and enzymatic effectors such as endolysins and holins [59,60].

Nonetheless, AlphaFold is less effective at predicting intrinsically disordered regions (IDRs)—amino acid stretches that lack stable tertiary structures yet play pivotal roles in protein–protein and protein–RNA interactions. IDRs often exhibit low pLDDT (predicted local distance difference test) scores, rendering them difficult for AI models to accurately predict. Experimental techniques such as small-angle X-ray scattering (SAXS), nuclear magnetic resonance (NMR), and cryo-electron microscopy (cryo-EM), along with simulation-informed hybrid modeling, remain essential to resolving the conformational ensemble of IDRs [61]. To address the structural diversity in bacteriophages, Klein-Sousa et al. [60] conducted a comprehensive mapping of phage tail fiber proteins using AlphaFold-Multimer v2.3.1, revealing conserved and novel folds across viral taxa. The study cleverly integrated pLDDT filtering with composite cryo-EM reconstructions to validate predicted architectures and identify IDRs. Recent efforts are assembling large-scale surveys of phage structures, but the field remains nascent and will require systematic experimental validation [60].

Together, these AI-augmented approaches are enhancing phage structural biology by providing more detailed predictions of phage structures, yet these predictions still require further experimental validation (e.g., cryo-EM, NMR, SAXS) before they can be considered dynamic and mechanistic.

### 3.2. Interface Modelling and Functional Inference

Since protein structures and genomic organizations have been delineated, the subsequent focus is now on functional roles and host specificity. To this end, a growing suite of AI-driven frameworks now enables the prediction of phage–host interactions and the functional annotation of previously uncharacterized genes. For example, AI-enabled docking with AutoDock Vina and Rosetta accelerates functional annotation by predicting phage protein–host interactions [62,63]. Docking showed that *Pseudomonas* phage LUZ19 AcrF1 competitively inhibits the Cas3 ATPase, guiding CRISPR-resistant phage cocktail design. DeepGO-SE further refines functional predictions from peptidoglycan hydrolysis to viral replication regulation [64].

Evolutionary scale modeling (ESM), an AI-developed pretraining models for protein sequences, has also shown great promise. GOPhage utilizes ESM2 embeddings combined with Transformer models (a neural network architecture) to improve Gene Ontology (GO) term predictions and annotate previously uncharacterized proteins [65]. DepoScope fine-tunes ESM2 on depolymerases and adds convolutional layers for simultaneous classification and domain boundary prediction; ESMFold validation confirmed that 78 of 123 candidates adopted recognized depolymerase folds [66]. Meanwhile, GSPHI links structural deep network embedding with deep neural networks to predict phage–host interactions [67]. It achieved an accuracy rate of 86.7% and an area under the curve (AUC) score of 0.9208, indicating its effectiveness in predicting phage–host interactions. A CRISPR-interference (CRISPRi) screen in *Mycobacterium smegmatis* identified host genes essential for phage ADS1 replication, including lipid biosynthesis enzymes crucial for envelope assembly [68].

Collectively, these advanced machine learning tools represent a high-quality and efficient advance in decoding phage protein function and host specificity, offering mechanistic insights into phage biology.

### 3.3. Multi-Omics Integration

Recent developments in multi-omics integration, which combines genomics, transcriptomics, proteomics, and metabolomics, are providing unprecedented insights into phage–host interactions at the systems level. These integrated approaches illuminate how phages modulate bacterial physiology, metabolism, and gene expression, offering insights critical for therapeutic design, ecological modeling, and industrial microbiology. For instance, Cucić et al. [69] leveraged integrative omics to characterize the infection program of phage CKA15 against *Listeria monocytogenes*, revealing novel regulatory and metabolic perturbations. To support such complex analyses, platforms such as KBase combine an object-oriented data model with a user-friendly Narrative interface to support the assembly, annotation, simulation, and sharing of microbial and community-scale models, effectively overcoming data silos and tool fragmentation [70]. Complementary to this, MetaPhage—built on the Nextflow architecture—orchestrates scalable, containerized workflows for automated detection, classification, and annotation of phages from metagenomic datasets, producing interactive reports amenable to downstream AI-based inference [71]. Together with emerging deep learning tools such as GOPhage and DepoScope, these platforms constitute an increasingly comprehensive and interoperable toolkit for phage systems biology.

Recent studies further highlight the utility of metabolomic profiling. For instance, infection by *Pseudomonas* phage LUZ19 was found to suppress *argH*, disrupting arginine biosynthesis and inducing auxotrophy—rendering the bacteria more vulnerable to nitric oxide-based immune defense mechanisms [72]. Meanwhile, workflows integrating cryo-EM imaging with omics analyses are now isolating and resolving structural features of phages directly from environmental samples [73]. Other studies have applied these frameworks to fermented food ecosystems, where integrated omics uncovered phage-mediated modulation of microbial communities and metabolic fluxes in the Daqu microbiome [74]. Additionally, population-specific microbiome studies are beginning to incorporate phage abundance and functional potential as part of host-specific disease susceptibility analyses [75].

Taken together, these advances in multi-omics, structural biology, and pipeline engineering now form the foundation for rational phage engineering. While linking molecular-scale dynamics to systems-level outcomes offers promising avenues for microbiome interventions, the application of AI in synthetic biology and phage therapy still faces significant technical hurdles that need further investigation and validation.

**Table 1:** AI-Driven phage structural and functional annotation tools.

Tool	Function	Input Data Type	Application	Refs.
Alphafold, Alphafold2	Protein 3D structure prediction	Amino acid sequences	Prediction of *E. coli* phage T4 tail fiber structures (β-helix domains)	[16,17,53]
AutoDock Vina	Molecular docking simulations	Receptor/ligand 3D models	Binding mechanism of *Pseudomonas* phage LUZ19 AcrF1 with Cas3 nuclease	[64]
Rosetta	Protein-host interaction interface optimization	Mutant libraries	Substrate-binding cleft optimization of *Streptococcus* phage PlyC endolysin	[65]
DeepLysin	Mining cryptic lysins from unannotated ORFs	ORF sequences	Discovery of LLysSA9 (41.2% validation rate)	[14]
DeepGO-SE	Protein functional annotation (Gene Ontology)	Sequences + ESM2 language model	Prediction of peptidoglycan hydrolysis activity	[66]
GOPhage	Phage protein function prediction using genome context	Genomic protein sequences + ESM2 embeddings	GO term annotation and identification of cryptic holins	[67]
DepoScope	Functional domain annotation of phage depolymerases	ORF sequences + structural language model	Domain-level prediction and 3D structure validation of depolymerases	[68]
GSPHI	Phage–host interaction prediction	DNA sequences + tail proteins + host receptors	ESKAPE pathogen host range prediction (AUC = 0.9208)	[70]
KBase	Multi-omics data integration platform	Cross-omics data	Standardized phage–host interaction analysis (metabolomics + transcriptomics)	[71]
MetaPhage	Modular pipeline for metagenomic phage annotation	Metagenomic reads	Scalable detection, classification, and reporting of phages from environmental datasets	[72]

## 4. Engineering Strategies for Phage Customization

### 4.1. Genome Modularization and Synthetic Phage Design

AI now enables the elucidation of functions and structures for previously uncharacterized bacteriophage genes. However, translating these discoveries into practical applications necessitates advanced genome editing technologies, with CRISPR-Cas systems playing a pivotal role. CRISPR-Cas systems employ RNA-guided endonucleases, proteins that precisely cleave DNA at programmable target sequences specified by guide RNA (gRNA). Significant advancements in AI-driven tools for predicting gRNA efficacy have now surpassed traditional methods. These tools offer researchers enhanced accuracy and speed in selecting optimal gRNAs. Innovations such as Cas-OFFinder, DeepCpf1, and Apindel further refine the prediction of CRISPR system off-target effects and on-target efficacy [76,77,78], ensuring more reliable and precise gene editing outcomes. Collectively, these developments streamline the design of highly specific gRNAs, minimize off-target effects, and maximize genome editing success.

Recent advances in CRISPR-Cas genome editing have revolutionized synthetic phage engineering, enabling precise host range expansion and efficient integration of therapeutic payloads [79]. Contemporary synthetic biology now harnesses CRISPR ribonucleoprotein (RNP) complexes for multiplexed editing, facilitating the modular assembly of functional cassettes—including virulence factor suppressors, biofilm-degrading enzymes, and antibiotic payloads [80]. Past-CRISPR, introduced by Li et al., represents a major leap in editing fidelity; by reducing mosaicism and enhancing allele-specific efficiency in complex genomes, it is readily adaptable to bacteriophage systems [81]. This approach partitions phage genomes into modular components (capsid, replication, lysis, payloads), allowing rapid host-specific reprogramming [20,82]. Multiple investigations affirm this paradigm. A recent report showcased CRISPR-based reprogramming of T-series phages to broaden host specificity [83], while high-throughput anti-CRISPR protein engineering now affords refined control over phage behavior and editing outcomes [84]. Complementing these advances, a dual-selection platform evolved riboswitch-regulated T7 phages that achieved a 28% gain in activation efficiency under alternating theophylline selection, converging on optimized motifs such as TTGCATCG [85]. These findings support using CRISPR-edited phages to precisely control complex microbiomes.

Beyond genomic rewiring, engineered virions can couple and deliver various functional materials, such as antimicrobial peptides [86], small-acid soluble spore proteins (SASPs) [87], addiction toxins [88], and redesigned transcriptional regulators [89]. Such payloads incapacitate alarmone signaling, halt septum formation, and disrupt DNA replication and protein synthesis. When armed with restriction endonucleases or holins [90], they rapidly dismantle chromosomal integrity and membrane potential, slashing bacterial burdens by orders of magnitude within hours. Complementing these biochemical assaults, photothermally active “phanorods”—chimeric phages conjugated to gold nanorods—enable near-infrared-triggered, highly selective ablation of bacteria, even within recalcitrant biofilms; irradiation simultaneously inactivates the phages themselves, curtailing unintended replication or gene transfer [91]. Collectively, programmable editing, directed evolution, and therapeutic phage assembly now converge to construct a versatile framework for next-generation antimicrobials amid escalating antibiotic resistance.

### 4.2. Receptor-Binding Protein (RBP) Engineering

Receptor-binding proteins (RBPs), primarily tail fibers, mediate the initial recognition and attachment of bacteriophages to specific bacterial hosts [92]. Engineering these RBPs can broaden or narrow host specificity, significantly influencing therapeutic applicability. Traditional methods for RBP engineering, including directed evolution through random mutagenesis and rational design. For instance, Otsuka et al. [93] employed a mutagenesis-driven approach targeting the distal tip of the gp37 tail fiber protein in T4 phages. By screening a mutant library against alternative receptors—including the OmpC receptor of pathogenic *E. coli* O157 and lipopolysaccharides of *E. coli* K12—mutants capable of efficiently binding these new receptors were isolated [93]. Similarly, Lu and colleagues performed targeted mutagenesis on host-range-determining regions of phage T3 tail fibers, successfully creating variants that could infect previously resistant bacterial mutants [94]. These approaches rely on prior genetic and structural knowledge to direct mutations towards regions predicted to significantly influence host interactions, enhancing the likelihood of isolating beneficial variants from finite libraries. However, specific knowledge of critical residues is not always essential for success. Dunne et al. illustrated this by randomly mutagenizing the RBP (Gp15) of *Listeria* phage PSA, leading to the isolation of mutants with broadened host specificity [95]. To streamline the selection of optimized variants, innovative selection platforms such as GOTraP (general optimization of transducing particles) have been developed [96]. GOTraP physically couples phage phenotypes (e.g., altered tail fibers) with their genotypes, facilitating rapid isolation of desired host range variants through efficient transduction screening.

Complementing directed evolutionary strategies, rational design methodologies leverage high-resolution structural insights (e.g., cryo-EM, SAXS) and bioinformatics to engineer bacteriophages with tailored receptor-binding proteins (RBPs). A key success lies in constructing chimeric RBPs through functional domain swapping between distinct phages. For instance, Tanji et al. utilized CRISPR/Cas9 editing to combine *E. coli* phages PP01 and T2 RBPs, achieving altered host recognition, albeit with reduced infectivity [83]. These approaches critically benefit from precise structural determination to identify functional domain boundaries and receptor interaction sites. Notably, structural biology faces challenges in resolving elongated, flexible trimeric proteins such as phage tail fibers. This limitation is increasingly addressed by AI-driven structural prediction tools such as AlphaFold and ESMFold, which significantly improve full-length tail fiber modeling accuracy and efficiency [60].

Recent studies expanded the engineering toolkit with data-driven and modular strategies. Wang et al. [97] analyzed 3021 phage–host lysis interactions across 238 *Klebsiella pneumoniae* strains, identifying six RBP clusters associated with specific capsular serotypes and demonstrating tunable capsule tropism through RBP exchange. Similarly, Yehl et al. used deep mutational scanning of tail fibers to generate phage variants that suppress resistance and access broader host ranges [94]. Modular assembly platforms such as VersaTile allow rapid recombination of RBP domains to retarget phages toward *E. coli* and *K. pneumoniae* [98]. Together with machine learning tools such as CHOOSER and structure-guided mutagenesis, they may support a scalable path to programmable tropism phages, advancing therapeutic optimization and contributing to clearer regulatory frameworks [82].

### 4.3. Lytic Enzyme Optimization

Endolysins are peptidoglycan-degrading enzybiotics that lyse bacterial cells from within during phage replication, while depolymerases (including virion-associated lysins) function extracellularly to dismantle surface polysaccharides and facilitate phage DNA injection [99,100]. Engineering these enzymes—adding membrane-permeabilizing peptides and rearranging domains—allows penetration of Gram-negative outer membrane barriers with potent bactericidal activity [101]. Their modular architecture enables rational tuning of catalytic potency, host range tropism, and pharmacokinetics, supporting therapeutic use against multidrug-resistant pathogens. For instance, Chandran et al. engineered recombinant lysins by fusing an endolysin and a virion-associated peptidoglycan hydrolase (VAPGH) to the SPK1 signal peptide; against MDR *S. aureus*, Endo88 outperformed VAH88 [102]. In *Clostridioides difficile*, engineered depolymerases digested polysaccharide capsules, sensitizing bacteria to phage attack and host immunity [103]. The arti-lysin AL-3AA—endolysin LysPA26 fused to the antimicrobial peptide SMAP29 by a three-amino-acid linker—rapidly lysed *P. aeruginosa* biofilms and showed broad activity against *Klebsiella pneumoniae* and *Escherichia coli* [104]. Likewise, unPEGylated cationic carbosilane dendrimers, although already potent, eradicated *P. aeruginosa* even more effectively when combined with phage-derived endolysins, which disrupt the outer membrane and expose peptidoglycan [105].

Building upon these engineering innovations, AI-driven platforms now expedite the discovery and refinement of phage lytic enzymes. DeepLysin exemplifies this advancement, employing a stacked machine learning model to mine cryptic endolysins from unannotated open reading frames (ORFs), achieving ~41% experimental validation (7 active lysins identified from 17 predictions) [14]. Similarly, the convolutional neural network framework DeepMineLys screened >370,000 microbiome-derived proteins, pinpointing ~18,500 putative endolysins—including novel enzymes demonstrating muralytic activity up to 6.2-fold greater than hen egg white lysozyme in vitro assays [106]. Beyond specialized lysin mining pipelines, comprehensive deep learning design frameworks play a monumental role in bridging computational prediction and experimental validation. Zimmerman et al. [107] recently developed CoSaNN, a context-dependent workflow integrating AlphaFold2 backbone sampling, ProteinMPNN sequence optimization, and SolvIT graph neural network solubility filtration. Within one design iteration, 54% of 348 chimeric enzymes achieved soluble expression in *E. coli*, with over 30% exhibiting higher melting temperatures than parental scaffolds—all accomplished without high-throughput screening.

### 4.4. Synthetic Phage Consortia

Rationally designed cocktails unite phages with complementary host ranges and lytic mechanisms to curb resistance. In the conventional, empirically driven workflow, individual phages are screened and then mixed; for example, a five-phage cocktail against *Escherichia coli* and a three-phage cocktail against *Staphylococcus aureus* reduced bacterial loads in milk models by 30–45% and suppressed biofilm formation by 50–99% [108]. Likewise, adaptive cocktails eradicated *Clostridioides difficile* in vivo while sparing commensal microbiota [103]. Engineering can further amplify the potency of such consortia. Gencay et al. armed phages with CRISPR-Cas payloads and optimized their tail fiber receptor-binding domains, generating SNIPR001—a cocktail that efficiently targets *E. coli*, including multidrug-resistant strains, and demonstrates robust in vivo efficacy and microbiome safety, thereby offering a promising alternative to prophylactic antibiotics [109]. Moreover, machine learning pipelines now markedly accelerate cocktail optimization [15] and will facilitate the rational selection of future engineered phage combinations.

Together, genome modularization, RBP engineering, lytic enzyme optimization, and synthetic consortia collectively provide a flexible foundation for phage customization. When combined with AI-based prediction, these strategies support more controlled host range modulation and therapeutic payload delivery.

## 5. Therapeutic and Biotechnological Applications

Enhanced by gene editing tools and AI, engineered bacteriophages demonstrate substantial therapeutic and biotechnological potential (Table 2). This section examines their applications in medicine, microbiome modulation, biosensors, agricultural biocontrol, and environmental remediation.

### 5.1. Combating Multidrug-Resistant Infections

Resistance in ESKAPE pathogens—*Enterococcus faecium*, *Staphylococcus aureus*, *Klebsiella pneumoniae*, *Acinetobacter baumannii*, *Pseudomonas aeruginosa*, and *Enterobacter* spp.—can arise rapidly via diverse mechanisms, including target site mutations in topoisomerases (e.g., *gyrA*, *parC*), upregulation of multidrug efflux pumps with reduced porin permeability (e.g., decreased OmpF), and the spread of mobile β-lactamases that compromise even newer agents [110]. In this context, engineered bacteriophages have emerged as precision antimicrobials against these organisms, whose escalating resistance undermines both established and next-generation antibiotics [111].

Proof-of-concept studies illustrate how phage platforms might complement existing therapies. Synthetic phages tailored to penetrate *S. aureus* biofilms unite CRISPR-Cas modules that excise resistance genes with biofilm-degrading enzymes such as DNase I and alginate lyase, eliminating more than 84% of biomass [112]. The AI-discovered phage lysin LLysSA9 eradicates Methicillin-resistant *Staphylococcus aureus* (MRSA) within ten minutes and maintains efficacy without inducing resistance, even after extended exposure [14]. A landmark compassionate use case employed three engineered phages—Muddy, ZoeJΔ45, and BPsΔ33HTH-HRM10—to treat disseminated, drug-refractory *Mycobacterium abscessus* in a 15-year-old cystic fibrosis patient, rapidly improving wound healing, hepatic function, and cutaneous lesions without adverse effects; intravenous delivery of the optimized cocktail sustained pathogen suppression and in vivo phage replication, heralding personalized therapy for resistant mycobacterial infections [113] and spurring ongoing clinical trials in Europe and the United States.

Phage–antibiotic synergy (PAS) augments this arsenal; subinhibitory β-lactams or quinolones induce bacterial fila mentation via division arrest and SOS activation, thereby increasing phage adsorption, accelerating lysis, and expanding burst sizes [114,115]. Beyond their bactericidal capacity, engineered phages can be tailored to reprogrammed bacterial physiology, thereby desensitizing pathogens to antibiotics or dampening their virulence. By delivering dominant drug-sensitive alleles, they overwrite resistance determinants and restore susceptibility to specific antimicrobials [116]. Similarly, phage-mediated transduction and overexpression of cognate uptake channels—such as OmpF—augment membrane permeability, enhancing antibiotic influx [117]. The success of these synergistic strategies, however, rests on a nuanced understanding of the molecular circuitry governing each bacterium–antibiotic interplay.

### 5.2. Microbiome Modulation

Phage-guided genome editing offers a potential route to address dysbiosis, but its therapeutic use remains under active investigation. In experimental systems, engineered phage consortia responsive to quorum sensing or environmental cues have been shown to alter community composition. For instance, phage-mediated delivery of bile salt hydrolases restores secondary bile acid metabolism disrupted in inflammatory bowel disease (IBD), counteracting pathogenic microbial transformations and reestablishing the anti-inflammatory properties of bile acids typically compromised by excessive sulphation [118,119]. Similar approaches effectively mitigate metabolic disorders; targeted removal of *Escherichia coli* strains overexpressing the *ramA* efflux pump significantly reduces systemic inflammation and insulin resistance in murine models [120]. Remarkably, a lytic T4 phage derivative employing the gp22 promoter enabled sustained in situ delivery of therapeutic proteins within the mammalian gut. By infecting resident *E. coli* to produce serpin B1a—an inhibitor of neutrophil elastase—the engineered phage alleviated colitis symptoms. Concurrently, delivery of *ClpB* promoted satiety signaling, mitigating diet-induced obesity, marking the first instance of successful in vivo therapeutic protein release mediated by lytic bacteriophages in mammals [121].

### 5.3. Phage-Based Biosensors

Phage-derived biosensors now furnish rapid, highly specific pathogen detection in industrial and environmental settings. A gold nanoparticle sensor functionalized with two *S. aureus*-binding peptides (pep23, pep28) attained a detection limit of 2.35 CFU mL^−1^ via colorimetric amplification [122]. Photonic-integrated circuit devices coated with M13 phages detected *Vibrio anguillarum* in aquaculture at 44.9 pfu mL^−1^, enabling early outbreak alerts [123]. Advanced immobilization strategies—including covalent grafting, phage display, and encapsulation within alginate hydrogels—enhance sensor durability and reusability under demanding conditions [124,125]. These platforms are now being adapted to intricate matrices such as municipal wastewater, where phage-based biosensors promise real-time tracking of pathogenic microbes [126]. Exploiting the innate host specificity of bacteriophages, such devices selectively detect viable bacteria within heterogeneous microbial consortia, furnishing an indispensable surveillance layer for public health monitoring and wastewater epidemiology.

### 5.4. Biocontrol in Agriculture and Environment

In tobacco cultivation, a CRISPR-engineered filamentous phage (RSCqCRISPR-Cas) targeting the *hrpB* virulence gene of *Ralstonia solanacearum* boosted plant survival by 59.2% in infected soils [127]. Commercial successes include Omnilytics’ AgriPhage formulations (e.g., P-10 for *Pseudomonas syringae* in spinach and T-20 for tomato bacterial wilt), which curtail pesticide use without sacrificing yield, and EcoPhage’s GoldenEco cocktail, now deployed at scale against tomato and pepper pathogens in Brazil [128]. Recombinant phages evolved in vitro can even overcome phage-resistant *Listeria monocytogenes*, expanding the toolkit for food safety and agricultural biocontrol [129].

Expanding beyond agricultural applications, CRISPR-Cas9 has also been proposed as a next-generation strategy for managing microbial infections in aquaculture. As outlined in recent work, this tool enables precise genetic editing of pathogenic bacteria, promotes disease resistance in aquatic species, and can be coupled with phage therapy or microbiome engineering to restore ecological balance [130]. These approaches promise to reduce antibiotic use in fish farming while safeguarding environmental health. Additionally, engineered phages and rationally formulated cocktails are becoming critical for precise microbial control in water and wastewater treatment systems [131].

By integrating CRISPR payloads, sophisticated encapsulation and synergistic biological agents, phage-based technologies have the potential to enhance soil health and bolster crop performance. Coupled with AI-driven phage optimization and adaptive regulatory frameworks, these innovations could contribute to more sustainable agriculture while helping to limit environmental impacts and the spread of antimicrobial resistance.

## 6. Challenges and Future Perspectives

Despite the tremendous potential of AI in phage research, challenges remain, particularly with high rates of false-positive predictions, the inaccurate prediction of intrinsically disordered regions (IDRs), and concerns over horizontal gene transfer (HGT) in AI-generated phages. These issues require careful validation through experimental methods and the integration of multi-omics data for more robust predictions.

AI models now routinely assign putative structures and functions to thousands of previously unannotated phage proteins. However, high rates of false positives and functional misassignments underscore the need for robust experimental confirmation. Innovations such as AI-enhanced microscopy and single-cell phenotypic modeling [132] are beginning to resolve infection heterogeneity at unprecedented resolution. Incorporating Bayesian neural networks and uncertainty-aware prioritization tools could streamline candidate triage processes, significantly reducing downstream experimental overheads. While AlphaFold2 excels in modeling well-folded protein domains, it continues to falter when confronted with intrinsically disordered regions (IDRs) and non-canonical chemistries such as D-amino-acid motifs, which are frequently implicated in host subversion or immune modulation [133]. Recent work [134] demonstrates how multi-scale AI modeling, informed by cryo-EM and generative language models, may overcome these blind spots and enable the design of flexible, bioactive phage scaffolds.

The inherent capacity of phages to facilitate horizontal gene transfer (HGT) raises valid biosafety concerns, particularly regarding the spread of antibiotic resistance genes (ARGs). Surveys of wastewater biomes [9] reveal widespread phage-mediated ARG dissemination. To mitigate this risk, AI-predicted phages must be equipped with synthetic safety mechanisms—such as CRISPR-based kill switches, genome watermarking, and irreversibly inactivated pay loads—alongside integration into global genomic surveillance frameworks. While graph-based models such as GSPHI and DeepGO-SE already achieve high predictive accuracy by integrating sequences and structures, truly context-aware models must incorporate temporally resolved omics data. Recent efforts to fuse digital phenotyping, transcriptomic flux, and metabolic rewiring point toward a systems-level view of phage–host dynamics—essential for rational design in clinical or ecological settings [132,135].

Static phage cocktails are inherently limited by bacterial evolutionary plasticity. Advances in quorum sensing and thermogenetic regulation [136] now enable the creation of programmable, adaptive phages that respond to local cues. Embedding these constructs within agent-based models or digital twins allows for predictive modeling of spatiotemporal infection landscapes and dynamic therapy optimization. The path to clinical and industrial implementation necessitates harmonized Good Manufacturing Practices (GMP)-compliant production, stringent QC pipelines, and detoxification protocols. Novel materials, such as endotoxin-free phage nanostructures [134], are emerging as scalable solutions. At the governance level, frameworks for AI-engineered phages—especially those featuring synthetic circuits or non-canonical residues—must evolve rapidly, guided by global pathogen intelligence systems and cross-jurisdictional biosafety standards [137].

If scientific, technical, and regulatory challenges can be addressed, AI-augmented phage engineering could enable the next generation of precision antimicrobials. Integrating advances in modular genome design, context-aware multi-omics, and programmable phage consortia may improve how we tackle antimicrobial resistance, manage complex microbial ecosystems, and develop living therapeutics—pending rigorous validation.

## 7. Conclusions

This review describes how artificial intelligence and synthetic biology have transformed bacteriophages from complex genetic structures into engineered antimicrobial agents. We survey recent applications of deep learning structure prediction, host range graph models, and modular genome editing to illuminate uncharacterized phage functions, map receptor-binding features, and prototype engineered virions equipped with CRISPR effectors, lysins, or quorum sensing modulators. Collectively, these developments support progress toward phage-based therapies for ESKAPE pathogens, biofilms, and microbiome-related disorders, while clinical use remains limited.

AI-driven multi-omics workflows are equally impactful, combining genomic, transcriptomic, proteomic, and metabolomic data to create computational models of phage–host interactions. These integrated systems support real-time tracking of infection pathways, condition-specific efficacy prediction, and the design of adaptive phage cocktails tuned to evolving microbiomes. Combined with new standards for purification, endotoxin removal, and genomic quality control, these advances enable scalable production and regulatory approval of AI-designed biologics.

Looking ahead, the field must resolve three intertwined challenges: (i) experimentally validating AI-predicted functions—especially within intrinsically disordered regions and non-canonical amino-acid scaffolds—through high-throughput screens and cryo-EM-guided molecular dynamics; (ii) implementing reliable safety controls to curb horizontal gene transfer while maintaining therapeutic potency; (iii) forging global, ethics-informed frameworks that democratize access to engineered phages without amplifying antimicrobial resistance. Success will hinge on tight collaboration between computational scientists, synthetic biologists, clinicians, and policy-makers, ensuring that phage innovation translates from silicon to bedside with speed, rigor, and equity.

## Figures and Tables

**Table 2:** Advances in phage engineering and associated AI tools.

Engineering Domain	Key Advances	AI Tools	Application Potential
Genome engineering	CRISPR-Cas-based expansion of host range through phage tail fiber editing [83] Integrated lysozymes, antibiotics, or anti-biofilm elements into phages [86,87,88,89] Enhanced editing fidelity through Past-CRISPR [81]	Cas-OFFinder [76] DeepCpf1 [77] Apindel [78] AlphaFold [16] CRISPR-RNP complexes [80]	Drug-resistant infection therapy Microbiome editing Biofilm penetration
Receptor-Binding Protein (RBP) Engineering	Rational design fused with directed evolution [93,94,95] Chimeric RBP design for expanded host range [83] High-throughput screening via GOTraP for optimized RBP functionality [96]	AlphaFold [16] ESMFold [66] CHOOSER [82]	Host range reprogramming Agricultural pathogen targeting Precision biofilm clearance
Lytic Enzyme Optimization	Endolysin and membrane-penetrating peptide fusion for Gram-negative bacteria [101] Fusion of peptidoglycan-binding domains to enhance biofilm penetration [102] Enzymatic activity mining [14,106,107]	DeepLysin [14] CoSaNN [107] DeepMineLys [106]	Outer membrane disruption Biofilm degradation Synergistic antibiotic therapy
Synthetic Phage Consortia	CRISPR-phage cocktails targeting multidrug-resistant pathogens [107] Dynamic regulation systems, such as riboswitches, to increase activation efficiency [102] Synergistic resistance suppression via multi-phage cocktails [108]	Cocktail selection machine learning model [15]	Safe gut microbiome intervention Dynamic therapeutic delivery Environmental pathogen control

## Data Availability

No new data were created or analyzed in this study. Data sharing is not applicable to this article.
